# Electroencephalography (EEG) in the Study of Equivalence Class Formation. An Explorative Study

**DOI:** 10.3389/fnhum.2017.00058

**Published:** 2017-03-21

**Authors:** Erik Arntzen, Hanna S. Steingrimsdottir

**Affiliations:** Department of Behavioral Science, Oslo and Akershus University CollegeOslo, Norway

**Keywords:** electroencephalography recordings, matching-to-sample, mild cognitive impairment, neurocognitive disorders, older adults, stimulus equivalence

## Abstract

Teaching arbitrary conditional discriminations and testing for derived relations may be essential for understanding changes in cognitive skills. Such conditional discrimination procedures are often used within stimulus equivalence research. For example, the participant is taught AB and BC relations and tested if emergent relations as BA, CB, AC and CA occur. The purpose of the current explorative experiment was to study stimulus equivalence class formation in older adults with electroencephalography (EEG) recordings as an additional measure. The EEG was used to learn about whether there was an indication of cognitive changes such as those observed in neurocognitive disorders (NCD). The present study included four participants who did conditional discrimination training and testing. The experimental design employed pre-class formation sorting and post-class formation sorting of the stimuli used in the experiment. EEG recordings were conducted before training, after training and after testing. The results showed that two participants formed equivalence classes, one participant failed in one of the three test relations, and one participant failed in two of the three test relations. This fourth participant also failed to sort the stimuli in accordance with the experimenter-defined stimulus equivalence classes during post-class formation sorting. The EEG indicated no cognitive decline in the first three participants but possible mild cognitive impairment (MCI) in the fourth participant. The results suggest that equivalence class formation may provide information about cognitive impairments such as those that are likely to occur in the early stages of NCD. The study recommends replications with broader samples.

## Introduction

Behavior analysts have approached the study of complex human behavior by using conditional discrimination procedures while teaching potential “if…then…” relations, followed by a test for additional untrained emergent relations (e.g., Sidman, 1994). Such procedures are often arranged in a matching-to-sample (MTS) format (Sidman and Tailby, 1982; Arntzen, 2006; Sidman, 2013). When establishing potential three 3-member classes (A→*B*→C), new relations could emerge during extinction conditions, that is, BA and CB (symmetry), AC (transitivity) and CA (equivalence; e.g., Sidman and Tailby, 1982). If the participant responds in accordance with the experimenter-defined stimulus classes when presented with all of the different stimulus pairs, the behavior is described as equivalence class formation. Several researchers have pointed out how emergent relations can contribute in the analysis of language and cognition (e.g., Barnes and Holmes, 1991; Sidman, 1994; Dougher et al., 2014). Also, studies have shown age differences in equivalence class formation (e.g., Saunders et al., 2005; Steingrimsdottir and Arntzen, 2014).

Cognitive skills often decline as a function of age (e.g., Kropotov et al., 2016). For example, Wilson and Milan (1995) demonstrated that yields on test for formation of equivalence relations were significantly lower among older adults when compared to the yields achieved by a group of younger participants. Similar data were found among healthy older adults (Gallagher and Keenan, 2009) and older adults with neurocognitive disorders (NCD; Bódi et al., 2009; Gallagher and Keenan, 2009). Moreover, Gallagher and Keenan (2009) noted that participants with NCD diagnoses were less likely to learn the baseline conditional discriminations prerequisite for the emergence of derived relations. They suggested that a stimulus equivalence paradigm may provide a sensitive measure for early changes in cognitive skills related to aging and NCD in older adults.

Furthermore, integrating additional measures while studying equivalence class formation may result in a more fine-grained analysis of the behavior as for example the use of eye-tracking technology (e.g., Dube et al., 2006; Hansen and Arntzen, 2015; Perez et al., 2015; Steingrimsdottir and Arntzen, 2016). Similarly, neuroimaging technologies such as functional magnetic reasoning imaging (fMRI) or electroencephalography (EEG) may help understanding how equivalence classes are formed. As Donahoe (1996) points out, “neural processes must be consistent with the orderly functional relations identified at the behavioral level, otherwise analyses at the neural level are either incorrect or incomplete” (p. 71).

A neuroimaging study focused on equivalence class formation by using fMRI measures to advance our understanding of potential mediating processes that may participate in the formation of stimulus equivalence classes (Dickins et al., 2001). Participants (mean age 27.6 years old) were exposed to a MTS training employing 18 iconic stimuli to form six 3-member equivalence classes. The results from the MTS test were compared to brain activation data obtained via fMRI. By comparing these data, the authors provided support to a linguistic basis for the formation of stimulus equivalence classes. However, they noted that as Broca’s area did not seem to be involved in the MTS, participants had probably not uttered, overtly or covertly, the name of the stimuli. Moreover, brain activation patterns showed that those participants who responded in accordance with stimulus equivalence “employed semantic mnemonic structures to mediate correct choice of comparison” (p. 5) which was in accordance with what the participants stated themselves in the debriefing of their study.

Another study which has focused on the neural mechanism involved in the formation of equivalence relations has included event-related potentials (ERP) measures (Wang and Dymond, 2013). The study arranged for conditional discrimination training of words and pseudowords in Experiment 1 and abstract stimuli in Experiment 2. The EEG recordings were done prior and after equivalence testing (in Experiment 2 only after equivalence testing), during a relatedness decision task. Hence, the participants (mean age 22.3 years old) were presented different prime-target stimulus pairs and instructed to decide whether or not they were related. The results showed that ERPs and the conditional discrimination training and testing correlated nicely. According to the authors, their results provided “further evidence of the functional anatomical correlates of a behavioral model of categorization” (p. 341).

Analyses of individual results using neural measures as EEG may contribute in the understanding of cognitive decline. Prince et al. (2011) emphasize that it is highly important to identify those who are at an early stage of a NCD, both for the informative value and for the possibility of doing intervention. One way of distinguishing between healthy older adults and those with early onset of NCD is to use biomarkers for the early detection of NCD, and EEG has already been used for that purpose (Rosén, 1997; Jeong, 2004; Snaedal et al., 2010; Jóhannesson et al., 2011; Micanovic and Pal, 2014; Engedal et al., 2015).

The rationale underlying the use of EEG for early detection of NCD is based on the hypothesis that most NCD cases are related to “disruption in the cholinergic neurotransmitter system” (Bartus et al., 1982, p. 408), and electrophysiology as measured by EEG is considered to be sensitive to such failure (e.g., Dringenberg, 2000). Moreover, the changes reflected in the resting state EEG have been studied in Snaedal et al. (2010) by contrasting elderly healthy controls with NCD participants. The study showed that applying multivariate statistical pattern recognition measures allowed quantitative separation of various stages of NCD, such as healthy controls, mild cognitive impairment (MCI) and Alzheimer’s disease (AD). Furthermore, results suggested that EEG may be used to identify MCI participants who are likely to develop clinical AD.

As noted by Donahoe (1996), neural processes should relate to behavioral data. Therefore, the purpose of the current study was to analyze stimulus equivalence class formation using EEG recordings as a supplementary measure. In addition, using the stimulus equivalence paradigm to study possible cognitive impairment in older adults.

## Materials and Methods

### Participants

Four female participants between 65–70 years (average age 65.8 years) voluntarily participated in the present experiment. They were informed before the beginning of the experiment that there was no compensation for their participation. All participants were Icelandic native speakers. The participants were recruited through personal contacts. Participants confirmed that they had no known problems with their vision and that they were right-handed. All participants signed an informed consent for their participation that declared their ability to withdraw from the study at any time. EEG recordings were made anonymous and participants were informed that they would not learn about their EEG results. Finally, participants were fully debriefed.

### Apparatus, Materials and Setting

**Figure 1:**
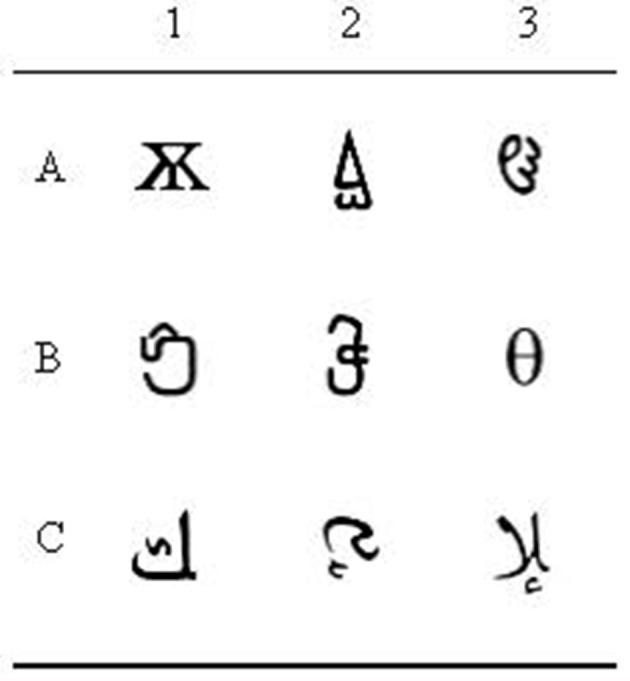
**The experimental stimuli**.

An HP laptop computer (Intel^®^ Core^TM^ i5 CPU M520@ 2.40 GHz) was used to present the arbitrary MTS training and testing. The MTS program gathered all the results from the conditional discrimination training and testing. Nine abstract shapes, chosen by the experimenter, were employed in the conditional discrimination training and testing (see Figure 1).

The EEG recording apparatus was a NicoletOne nEEG Module from VIASYS Healthcare Inc., Conshohocken, PA, USA. In order to conduct the EEG recording the experimenter used EEG electrodes, EEG conductive paste, Nuprep^®^ (Skin Prepping Gel) along with other products needed when placing the electrodes and for cleaning the participants’ hair afterward. The electrodes were placed on the participants’ scalp, and the gel functioned as a glue to keep electrodes on the scalp. Participants had to sit in the same position during the recording. The EEG results were uploaded to the MentisCura diagnostic server^1^ in Iceland for analysis. The analysis from MentisCura is provided in an “Ach Index”, developed by contrasting participants with an intact cholinergic system and participants with an impaired cholinergic system (Johnsen et al., 2014). The Ach Index is a scale ranging from 0 to 140, with the average index for a person with AD approximately 60, and 110 for healthy older adults. The lower the score, the more likely the person is identified as having MCI or AD.

The experiment was conducted in a bright, quiet room in a single session for each participant. In addition to the MTS computerized training and testing, and the EEG recordings, the participants answered the Mini–Mental State Examination (MMSE) questionnaire (Folstein et al., 1975), a quick assessment tool for cognitive functioning. The cutoff score is a lower score than 24. The participants in the present study had an MMSE score of 28 or above. Finally, the participants were handed laminated cards, approximately 3.5 × 5 cm in size for pre and post-class formation sorting of the stimuli used during the conditional discrimination training and testing.

### Procedure

Upon arrival at the experimental setting, the participant signed the informed consent form, answered the MMSE questionnaire, did a pre-class formation sorting of the stimuli where the experimenter handed the participant the deck of stimuli with the instruction “please sort these”, before being seated in front of the computer. There were three EEG recordings, one before the computerized training, one after the computerized training before testing, and finally, one after the computerized testing. The reason for comparing three EEG recordings was to examine any possible changes in the EEG after training and testing compared to baseline.

#### The Three EEG Recordings

The international standard 10–20 electrode placement system was used for the EEG recording with following positions: Fp1, Fp2, F3, F4, F7, F8, Fz, T3, T4, T5, T6, A1, A2, C3, C4, Cz, P3, P4, Pz, O1, O2 and Oz, with the Fpz as reference (Jasper, 1958; Snaedal et al., 2012). The three EEG recordings involved two consecutive intervals, starting with a 3-min eyes closed recording followed by a 3-min interval eyes open recording.

#### Computerized Arbitrary MTS Training and Testing

Before starting the computerized training and testing, the participant was presented with following written instructions:

“A stimulus will appear in the middle of the screen. Click on this by using the computer mouse. Three others will appear. Choose one of these by using the computer mouse. If you choose the stimulus we have defined as correct, words like very good, excellent and so on will appear on the screen. If you press the wrong stimulus, the word wrong will appear on the screen. At the bottom of the screen, the number of correct responses you have made will be counted. During some stages of the experiment, the computer will not tell you if your choices are correct or incorrect. However, based on what you have learned, you will be able to determine the correct response for each task. Please do your best to get everything right. Good luck. Press the start button to begin the test.”

The computer was set to run three phases: (1) acquisition of the baseline conditional discriminations; (2) maintenance phase; and (3) test phase. In the first two phases, the stimuli were presented in a many-to-one (MTO) training structure (training AC and BC trials). The MTO was employed because of producing high yield equivalence class formation (see Arntzen, 2012, for a discussion).

The training phase involved a 0-s delayed MTS. Hence, each training trial began with the presentation of the sample stimulus in the center of the screen. Upon clicking on the sample stimulus, the sample disappeared and was followed by the immediate appearance of three comparison stimuli. The comparison stimuli were presented randomly in different corners of the monitor, leaving one corner blank in each training trial. Following the participant’s response to one comparison stimulus, programmed consequences such as “correct” or “incorrect” were presented for 1500 ms. Each training trial ended with an inter-trial interval (ITI) where the computer screen was blank for 500 ms before another sample stimulus was presented.

Table 1 shows number of training trials in each block, the master criterion and probability of programmed consequences. The first phase started with the presentation of AC training trials (A1C1, A2C2 and A3C3). When the participant completed 14 out of 15 presentations correctly in one training block (five presentations of each trial type), BC training trials (B1C1, B2C2 and B3C3) were presented. When the participants completed 14 of 15 presentations correctly during the BC training trials, the AC and BC training trials were presented in a mixed training phase, with 30 training trials in each training block (five presentations of each trial type). During these first training phases, all trials ended with programmed consequences indicating whether the response was correct or incorrect. When participants reached the criterion of 90% accuracy in the second 30 trial block, the second phase (the maintenance phase) started. During the maintenance phase, the probability of programmed consequences was 75% in the first training block and reduced to 25% in the subsequent block, and finally to 0% in the last training block. For trials in which the programmed consequences were absent, the screen was blank for 2000 ms so that the length between the offset of a comparison stimulus and the onset of a sample stimulus would be equal, independent of whether programmed consequences were presented.

**Table 1:** **Trials per block and probability of programmed consequences**.

	Training phase	Trials/Block	% Probability of programmed consequences
**Baseline training**	A1C1, A2C2, A3C3	15	100
	B1C2, B2C2, B3C3	15	100
	A1C1, A2C2, A3C3, B1C2, B2C2, B3C3	30	100
**Maintenance**	A1C1, A2C2, A3C3, B1C1, B2C2, B3C3	30	75
	A1C1, A2C2, A3C3, B1C1, B2C2, B3C3	30	25
	A1C1, A2C2, A3C3, B1C1, B2C2, B3C3	30	0
**Test**	A1C1, A2C2, A3C3, B1C2, B2C2, B3C3, C1A1, C2A2, C3A3, C1B1, C2B2, C3B3, A1B1, A2B2, A3B3, B1A1, B2A2, B3A3	90	0

*Note: There were three conditional discrimination training and test phases: (1) baseline training; (2) maintenance; and (3) test. Probability of programmed consequences are shown in the right most column throughout each of the conditional discrimination phases*.

The final phase of the arbitrary MTS was a test phase (see Table 1), wherein the previously trained relations, AC and BC training trials, were interspersed between additional presentations of test relations; symmetry trials; CB and CA, and transitive/equivalence trials; AB and BA. The test was arranged as simultaneous MTS. The phase consisted of, in total, 90 test trials, 30 baseline trials, 30 symmetry trials and 30 equivalence trials. There were no programmed consequences during the testing phase. The accuracy criterion for passing a test for responding in accordance with stimulus equivalence was set to a minimum of 90% for each test trial type: the AC/BC trials, the symmetry trials and for the equivalence trials.

After the computerized training and testing and the final EEG analysis were complete, participants performed a post-class formation sorting of the stimuli, with the same laminated cards used in the pre-class formation sorting.

## Results

The results from the MMSE, the pre-class formation and post-class formation sorting, along with the computerized training and testing are shown in Table 2. None of the participants categorized the stimuli in accordance with the experimenter-defined classes during pre-class formation sorting, and all participants reported that their sorting was random.

**Table 2:** **The results from the MMSE and conditional discrimination training and test trials**.

*P*	MMSE	#AC/BC	Pre-cat.	AC/BC	SYM	EQ	Post-cat.
9571	29	645	No	**29**	**30**	**30**	Yes
9578	29	960	No	**28**	**29**	**27**	Yes
9573	30	330	No	**27**	**29**	21	Yes
9580	30	810	No	25	**27**	18	No

*Note: The numbers in bold indicate scorings according to the mastery criterion. P, participant; MMSE, Mini-Mental State Examination; #AC/BC, number of training trials before reaching mastery criterion; AC/BC, original training trials presented during the test; SYM, symmetry trials; EQ, equivalence Trials*.

**Figure 2:**
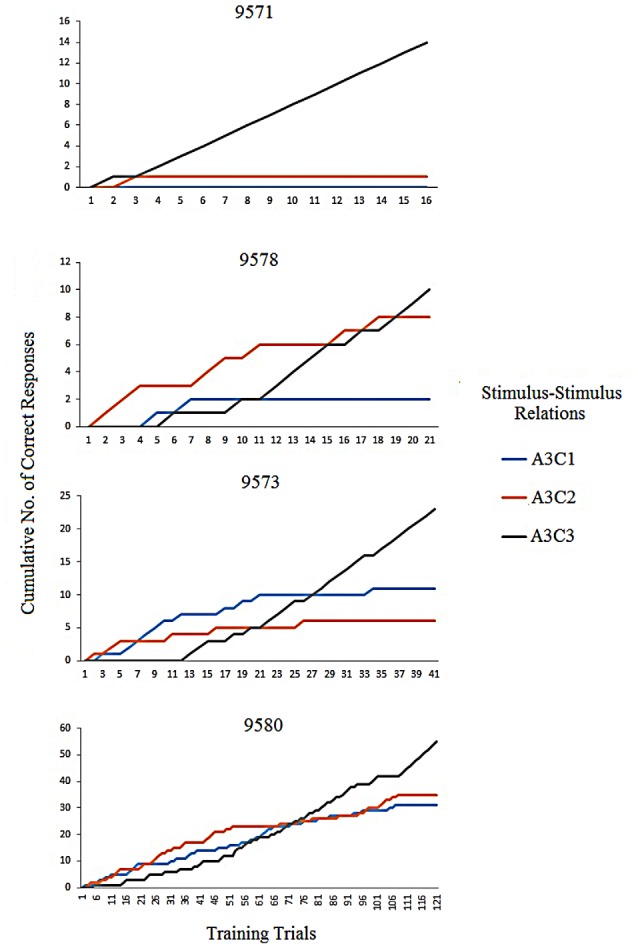
**The figure shows the individual cumulative records for the participants of correct responses while establishing one of the AC stimulus-stimulus relation; A3C3 is the correct choice and A3C1 and A3C2 are incorrect choices**.

An analysis of correct responses during the first training blocks of the conditional discrimination training showed that the effect of the differential reinforcement was slower in P9580 compared to the other three participants (P9571, P9573 and P9578). Figure 2 shows an example of the difference of the effect of differential reinforcement for the establishment of the A3C3 relation. A3C1 and A3C2 were followed by the programmed consequence “incorrect” which lead to the termination of responding to C1 and C2 in the presence of A3. A3C3 was followed by the programmed consequence “correct” leading to responding to C3 in the presence of A3. In a cumulative record, new responses are added on to the already existing total number of responses. Hence, the data line can only go up and steeper climb indicates a more rapid responding than slower climb. Results from the cumulative records show the establishment stimulus control by flattening out the A3C1 and A3C2 lines and incline in the A3C3 line. For P9580 the A3C3 relation was established after 108 trials, while for P9571, P9578 and P9573 the relation was established after three, 18 and 34 trials, respectively. P9580 received the highest number of programmed consequences on average before establishing the conditional discriminations. The participants’ responses were evenly distributed between all of the comparison stimuli. Hence, there was no indication of any preference of one stimulus.

**Figure 3:**
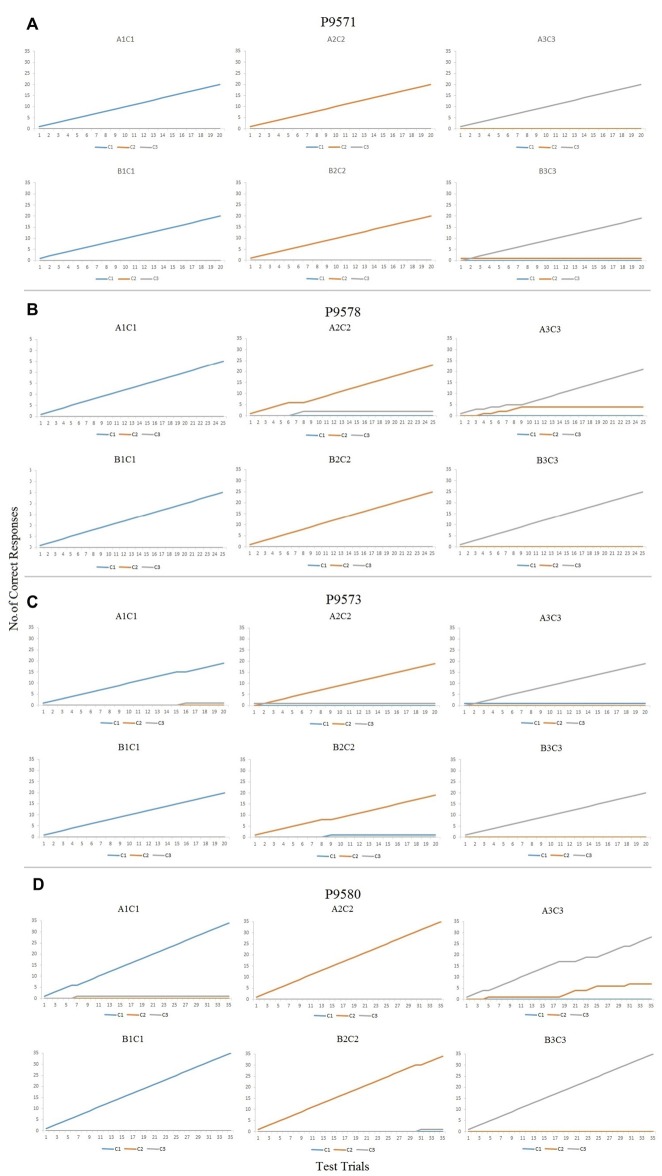
**(A)** Cumulative records for trials A1C1, A2C2, A3C3, B1C1, B2C2 and B3C3 showing correct responses during the maintenance phase for P9571. **(B)** Cumulative records for trials A1C1, A2C2, A3C3, B1C1, B2C2 and B3C3 showing correct responses during the maintenance phase for P9578. **(C)** Cumulative records for trials A1C1, A2C2, A3C3, B1C1, B2C2 and B3C3 showing correct responses during the maintenance phase for P9573. **(D)** Cumulative records for trials A1C1, A2C2, A3C3, B1C1, B2C2 and B3C3 showing correct responses during the maintenance phase for P9580.

Figures 3A–D (P9571, P9578, P9573 and P9580, respectively) show the cumulative numbers of correct responses during the maintenance phase of the conditional discrimination training. As can be seen in Figures 3A–C, the six baseline conditional discriminations trained in each participant were established in accordance to the experimenter-defined mastery criterion, with accurate responding throughout the maintenance phase (Table 1). P9580 also responded in accordance to the mastery criterion for demonstrating establishment of the baseline conditional discriminations (see Figure 3D). Noteworthy, although P9580 responded in accordance to the mastery criterion, the results in figure show that this participant made few errors when the likelihood of programmed consequences was reduced.

As Table 2 shows, P9571 and 9578 responded in accordance with stimulus equivalence, with 27 or more correct for each trial type. P9573 and P9580 did not respond in accordance with the mastery criterion for this experiment. P9573 responded with an accuracy lower than the mastery criterion for one of the derived relations, the equivalence trials, with 20 of 30 responses correct, whereas P9580 responded incorrectly on two types of test trials, the baseline probes AC/BC (26 of 30 correct) and the equivalence trials (17 of 30 correct), respectively.

**Figure 4:**
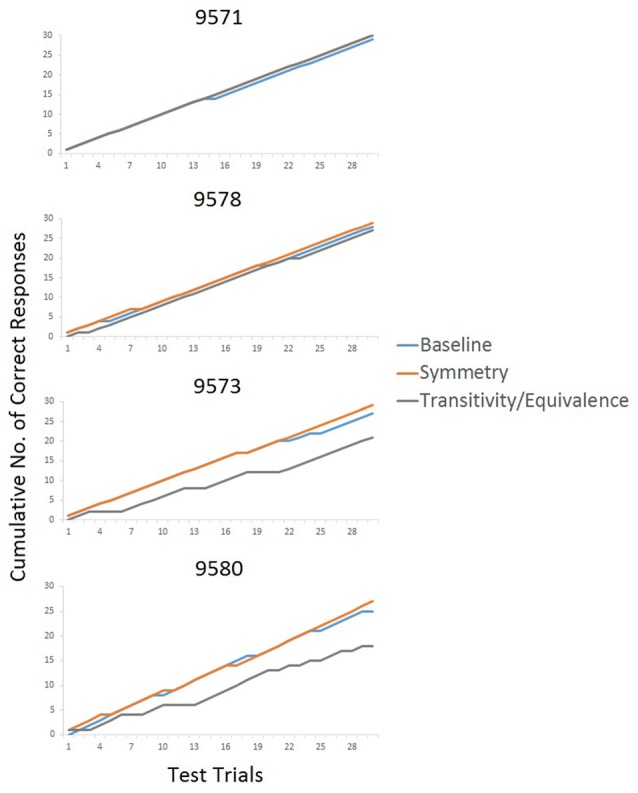
**Cumulative records showing each participant’s correct (shown with an incline of the line in the graph) and incorrect responses (flattening out of the line) during testing**.

Figure 4 provides fine-grained information about the participants’ performance during testing. The cumulative records for P9571 and P9578 show a steady incline with a great overlap between the three different test types, demonstrating correct responding on all test relations. In contrast, the cumulative curves for P9573 and P9580 show different degree of incline on the three tested relations, demonstrating the participant’s incorrect responses, particularly with changes in inclination of the transitivity/equivalence line. P9580 was the only participant who did not sort the stimuli in accordance with the experimenter-defined classes following arbitrary MTS training and testing. P9580 sorted Class 1 in accordance with the experimenter-defined class but showed a mixed sorting of Classes 2 and 3 in the post-class formation sorting (see Table 3).

**Table 3:** **Pre-class formation and post-class formation tests**.

Participant	Pre-class formation sorting test	Post-class formation sorting test
9571	111, 121, 101	**300, 030, 003**
9578	111, 121, 101	**300, 030, 003**
9573	555	**300, 030, 003**
9580	021, 210, 102	**300**, 021, 012

*Note: Sorting 300, 030, 003 represents correct sorting with (1) the three experimenter-defined stimuli sorted correctly in the first class and none in the second and third; (2) the three experimenter-defined stimuli sorted correctly in the second class and none in the first and second; and (3) the three experimenter-defined stimuli sorted correctly in the third class and none in the first and second*.

**Figure 5:**
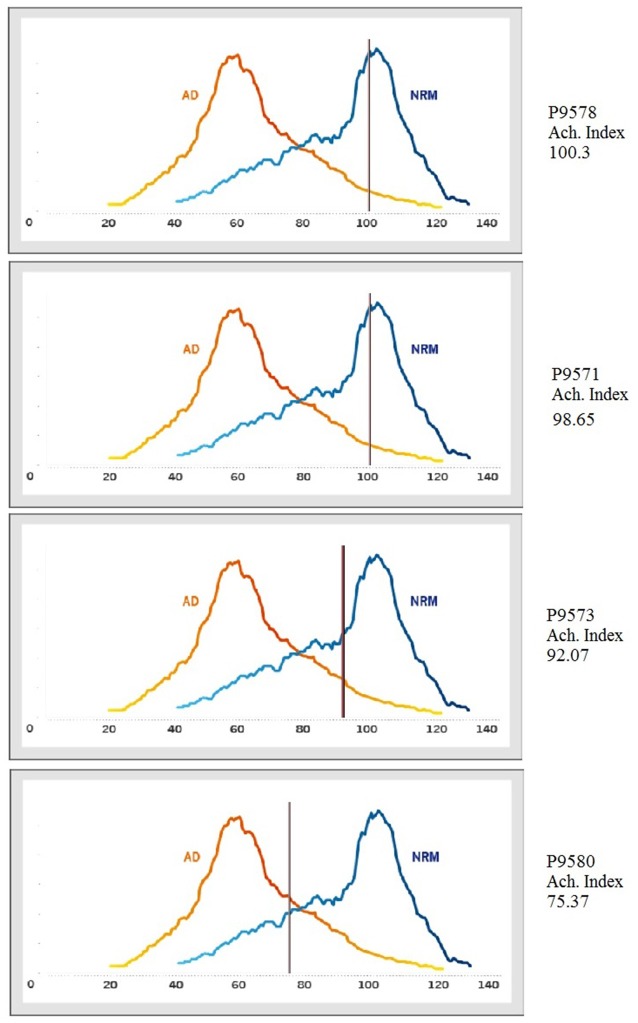
**Incidence frequency map for healthy controls, blue and Alzheimer’s disease (AD) participants, red.** The horizontal line (the Ach Index) on each curve shows the results from each participant in comparison to the results from the MentisCura database.

**Figure 6:**
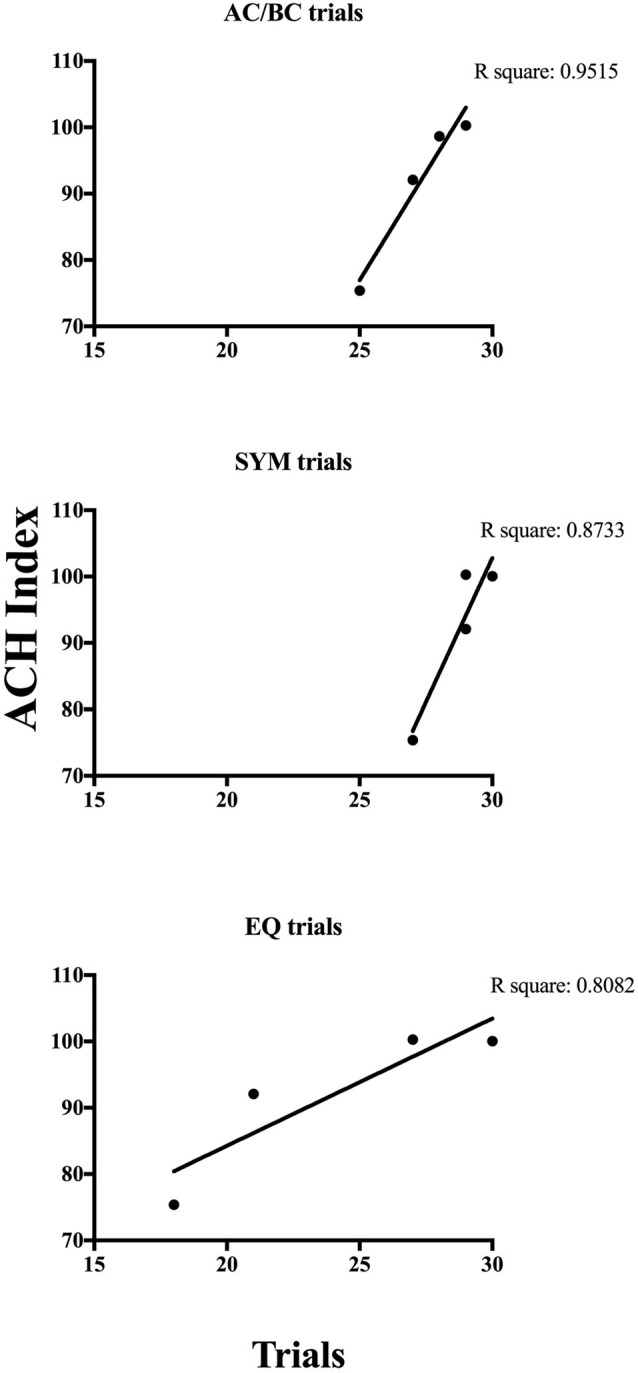
**Correlation analyses for AC/BC, symmetry (SYM) and equivalence (EQ) trials**.

According to the Ach Index retrieved from the EEG recordings taken in the current study (indicated by the horizontal line on each curve in Figure 5), P9571, P9573 and P9578 did not show indications of cognitive decline. However, it is noteworthy that comparing the incidence frequency of large healthy control cohorts shown in blue, and incidence frequency in large AD cohorts shown in red, shows that those participants who did not respond in accordance with stimulus equivalence (P9573 and P9580) came closer to the average of the AD group compared to those who passed (see the horizontal lines in Figure 5). Although P9573 did not meet the criterion for being diagnosed with MCI or AD, the EEG analysis for P9580 showed high consistency with MCI. The EEG recording was administered three times to each participant, i.e., before training, between training and testing and after testing, and the results from the EEG analysis were consistent for all three recordings. In addition, Figure 6 shows the correlation between behavioral data during testing and the ACH score. The correlation is highest for the performance on the AC/BC baseline trials, followed by performance on the symmetry trials and lowest on the equivalence trials in the test.

## Discussion

The purpose of the present explorative experiment was to study equivalence class formation in older adults using an EEG analysis as an additional measure for indication of possible changes in cognitive skills. As it may be difficult to differentiate individuals in the early stages of MCI from healthy older adults, the present study used EEG analysis, a method already in use for the early detection of cognitive decline, as an additional measure for cognitive changes.

The results showed that P9571 and P9578 responded in accordance with stimulus equivalence with no indications of possible NCD on the additional EEG measure. Therefore, these results confirm previous suggestions within this research area that the responding in accordance with stimulus equivalence reflects normal cognitive functioning (Barnes-Holmes et al., 2005; Dickins, 2005; Schlund and Cataldo, 2005; Haimson et al., 2009; Wang and Dymond, 2013; Bortoloti et al., 2014). P9573 did not respond in accordance with the experimenter-defined criterion for equivalence class formation. However, this participant’s EEG analysis did not show any signs of MCI. It is though noteworthy that this participant had a lower score on the Ach-Index compared to the P9571 and P9578. Hence, some issues need to be resolved in future studies. For example, it is important to look at where the accuracy criterion for responding in accordance with stimulus equivalence is set. If the accuracy criterion is set too high, the conclusion is made that the participant failed the stimulus equivalence test when he or she should have been considered to have passed the test. Conversely, if the accuracy criterion is set too low, the participant is considered to have passed the test although the stimulus equivalence classes were not properly established (see Arntzen, 2012, for a discussion).

The results from P9580 were notable as this participant failed on two of the test relations, the baseline probe trials and the equivalence trials. This participant also failed on the post-class formation sorting. Interestingly, the EEG indicated a high consistency with the MCI. Post-class formation sorting has been suggested to provide additional information regarding possible class establishment (e.g., Arntzen et al., 2015, 2017) and may thus be of utmost importance when studying stimulus equivalence class formation. These results may also indicate the possible necessity of focusing on more than one test relation when evaluating possible changes in cognitive skills.

Considering the training phase, P9573 completed the least number of training trials (330), which may have had such an effect on the stimulus class formation during testing that the participant failed the test. Therefore, the training criterion may be set higher or an additional training phase could be added to increase the minimum number of training trials before the test. Error analysis of the training phase for P9580 showed the greatest variation in responding, with the slowest establishment of the conditional discrimination relations in the beginning of training. Moreover, although the participant had responded in accordance to the accuracy criterion set during the initial training phases, P9580 made some incorrect responses when the density of programmed consequences was decreased. The effect of decreased density of programmed consequences during conditional discrimination training has been shown in an earlier study by Steingrimsdottir and Arntzen (2011). In the study the participant, a patient with dementia, made fewer correct responses when the density of programmed consequences was decreased. Hence, future studies may also consider the establishment and maintenance of conditional discrimination and possible relation with the onset of cognitive decline.

There are other procedural issues that need to be taken into consideration in future studies. For example, using Linear Series training structure (A→*B*→C) rather than the MTO may generate different results, as the former may not have ceiling effects to the same degree as the latter does. For this reason, future studies may show whether one type of training structure is more consistent with the EEG results compared with alternative training structures. Other methodological issues that may affect the likelihood of responding in accordance with stimulus equivalence that may require addressing have been discussed by Arntzen (2012).

Although the same experimental conditions were given to a small group of participants, we observed between-subject variability concerning the patterns of responding: two participants passed all the tests for equivalence relations, two participants demonstrated the emergence of two and one equivalence relations. In addition, one participant failed both the MTS test and the post-class formation sorting test. Each pattern of behavior on the MTS test may be related to a specific stage of NCD or to idiosyncratic patterns of responding inadvertently induced by MTS parameters (Iversen et al., 1986; McIlvane and Dube, 2003; Arntzen, 2012). Thus, we believe that the present study provides support for conducting further experiments in this area with a larger number of participants. The participants recruited for future experiments should include elderly whose score is between 27–30 on the MMSE, elderly who are being evaluated for the possible cognitive decline (MMSE score between 27–30 but are concerned about their health), and elderly that score between 23–27. Furthermore, future studies should include other neuropsychological tests as well to provide a fuller indication of the possible correlations between the likelihood of responding in accordance with stimulus equivalence and initial stages of cognitive decline. Additionally, the use of EEG for diagnosing NCD is promising (e.g., Rosén, 1997), though presently it is important to include other tests both to detect cognitive decline and to provide a more comprehensive understanding of the participant’s condition.

In conclusion, it has been suggested that behavior analysts could benefit from incorporating additional measures when studying behavior (Palmer, 2010). Moreover, according to Donahoe (1996), “In any science, phenomena are eventually encountered at the border between the level of analysis of that science and the neighboring sciences in which information from both sciences become crucial for understanding” (p. 72). Consistent with these statements, Ortu (2012) suggested that, under some circumstances, neuroscientific techniques may enable an experimenter to access some relevant variables that are frequently out of the scope of the traditional framework of behavioral analysis. At current date, there are only a few publications within the behavior analytic literature on the relationship between neural variables and studies on stimulus equivalence formation (e.g., Dickins et al., 2001; Barnes-Holmes et al., 2005; Haimson et al., 2009; Wang and Dymond, 2013). The current study contributes to this area by incorporating additional measures (specifically, EEG) for comparison with the behavioral measure. On a more wide-ranging level, the present study also underscores the possibility of using EEG when diagnosing NCD.

## Ethics Statement

Participants were treated in accordance with ethical standards of APA. All procedures performed in studies involving human participants were in accordance with the ethical standards and with the 1964 Helsinki Declaration and its later amendments or comparable ethical standards. Informed consent was obtained from all individual participants included in the study.

## Author Contributions

The authors have contributed to all the parts of the manuscript. The second author, HSS was the experimenter.

## Funding

This research was funded by Oslo and Akershus University College.

## Conflict of Interest Statement

The authors declare that the research was conducted in the absence of any commercial or financial relationships that could be construed as a potential conflict of interest.

## Footnotes

^1^http://www.mentiscura.com/
